# Localized Reaccumulation of Pericardial Effusion After Pericardial Fenestration Undetected on Frontal Chest Radiography

**DOI:** 10.7759/cureus.98311

**Published:** 2025-12-02

**Authors:** Chihiro Kobayashi, Shintaro Shimizu, Amano Yoshihiro, Kashu Kitani, Takeshi Isobe, Tamio Okimoto

**Affiliations:** 1 Division of Medical Oncology ＆ Respiratory Medicine, Department of Internal Medicine, Shimane University Faculty of Medicine, Izumo, JPN

**Keywords:** cardiac tamponade, chest x-ray, echocardiogram, pericardial effusion, pericardial fenestration

## Abstract

A 70-year-old man with advanced lung adenocarcinoma visited our hospital complaining of dyspnea. He was diagnosed with cardiac tamponade and underwent pericardial drainage. Pericardial effusion reaccumulated in 73 days, and right pericardial fenestration was performed. Although his respiratory condition worsened three weeks after the surgery, his chest X-ray showed little change. He died of respiratory failure the next day. Autopsy CT revealed heterogeneously reaccumulated pericardial effusion in the pericardial cavity, which was difficult to detect by frontal chest X-ray. An autopsy revealed adhesions around the fenestration site. Pericardial effusion may heterogeneously reaccumulate in the pericardial cavity due to adhesions. When the respiratory condition worsens even after fenestration for cardiac tamponade, lateral imaging, echocardiography, and chest CT should also be considered.

## Introduction

Pericardial effusion can result from various etiologies. Contemporary hospital cohorts show a shift toward iatrogenic complications, with malignancy, infectious/inflammatory disease, and mechanical cardiac events remaining important contributors [1,2]. Iatrogenic complications were associated with a relatively better outcome compared to tamponade induced by malignant diseases [1,2]. The therapeutic goals in pericardial tamponade are rapid hemodynamic stabilization and long-term prevention of fluid reaccumulation. Although Japanese guidelines for lung cancer weakly recommend pericardial sclerosis, pericardial fenestration is not explicitly endorsed owing to the lack of prospective data [3]. In contrast, a systematic review of malignant effusions demonstrated superior symptom relief and lower recurrence rates with surgical drainage compared with non-surgical modalities, with overall success rates of 93.3-100% and complication rates of 4.5-10.3% [4].

Historically, recurrence after fenestration has been regarded as relatively uncommon, with reported rates of approximately 10% [5]. However, the true incidence and underlying mechanisms remain uncertain. Here, we report a case of localized, loculated pericardial effusion after fenestration caused by adhesions at the fenestration site. Notably, the recurrence was not detectable on frontal chest radiography but was identified only by postmortem CT and autopsy.

## Case presentation

**Figure 1:**
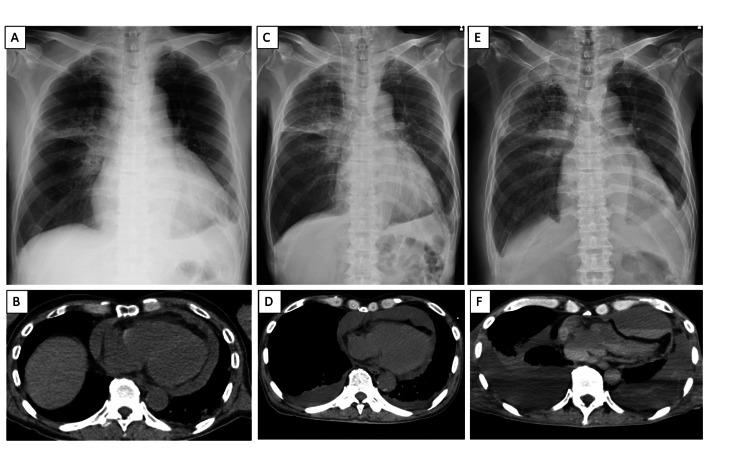
Chest radiography and CT findings. A. Frontal chest radiograph obtained in the sitting position at initial presentation, showing cardiomegaly compatible with a large pericardial effusion. B. CT at initial presentation demonstrating a large circumferential pericardial effusion with features of cardiac tamponade. C. Frontal chest radiograph (supine position) prior to pericardial fenestration. D. CT prior to pericardial fenestration revealing reaccumulation of pericardial fluid predominantly in the anterior pericardial space. E. Frontal chest radiograph (supine position) obtained three weeks after pericardial fenestration, showing neither cardiomegaly nor pulmonary abnormalities despite clinical deterioration. F. Postmortem CT performed several hours after image E, demonstrating a loculated pericardial effusion, which was not appreciable on frontal chest radiography.

A 70-year-old man with advanced lung adenocarcinoma presented with a one-week history of dyspnea. On admission, he was hypotensive and tachycardic. Echocardiography revealed a large circumferential pericardial effusion, consistent with cardiac tamponade, and urgent pericardial drainage was performed (Figures 1A, 1B). Cytological analysis of pericardial fluid was positive for adenocarcinoma, confirming malignant pericardial effusion. He was referred to our hospital for further investigation and treatment. He was initiated on a combination chemotherapy of carboplatin and pemetrexed; however, treatment was discontinued after two episodes of cerebral infarction and a subsequent decline in performance status. His symptoms initially improved; however, dyspnea recurred 73 days later. Frontal chest radiography showed no remarkable changes, but chest CT and echocardiography confirmed recurrent anterior pericardial effusion (Figures 1C, 1D). Right pericardial fenestration was therefore undertaken. The postoperative course was initially uneventful. Three weeks later, however, his respiratory status progressively declined. Frontal chest radiography demonstrated neither cardiomegaly nor pulmonary abnormalities (Figure 1E). At that time, his pulse rate increased to 120-140 beats per minute, and systolic blood pressure dropped below 80 mmHg. Oxygen saturation, which had been maintained at 97% with 3 L/min of oxygen the previous day, decreased to 85% despite an oxygen flow rate of 10 L/min. As the patient was receiving palliative care, no further diagnostic procedures were performed. Because of severe dyspnea, palliative sedation with midazolam was initiated. This clinical course was consistent with hemodynamic collapse due to recurrent cardiac tamponade and subsequent respiratory failure. The patient died the following day. Postmortem CT, performed several hours after the last chest radiography, revealed heterogeneously distributed pericardial fluid with septations localized to the left anterior and posterior pericardial spaces, which had not been visualized on chest radiography (Figure 1F).

**Figure 2:**
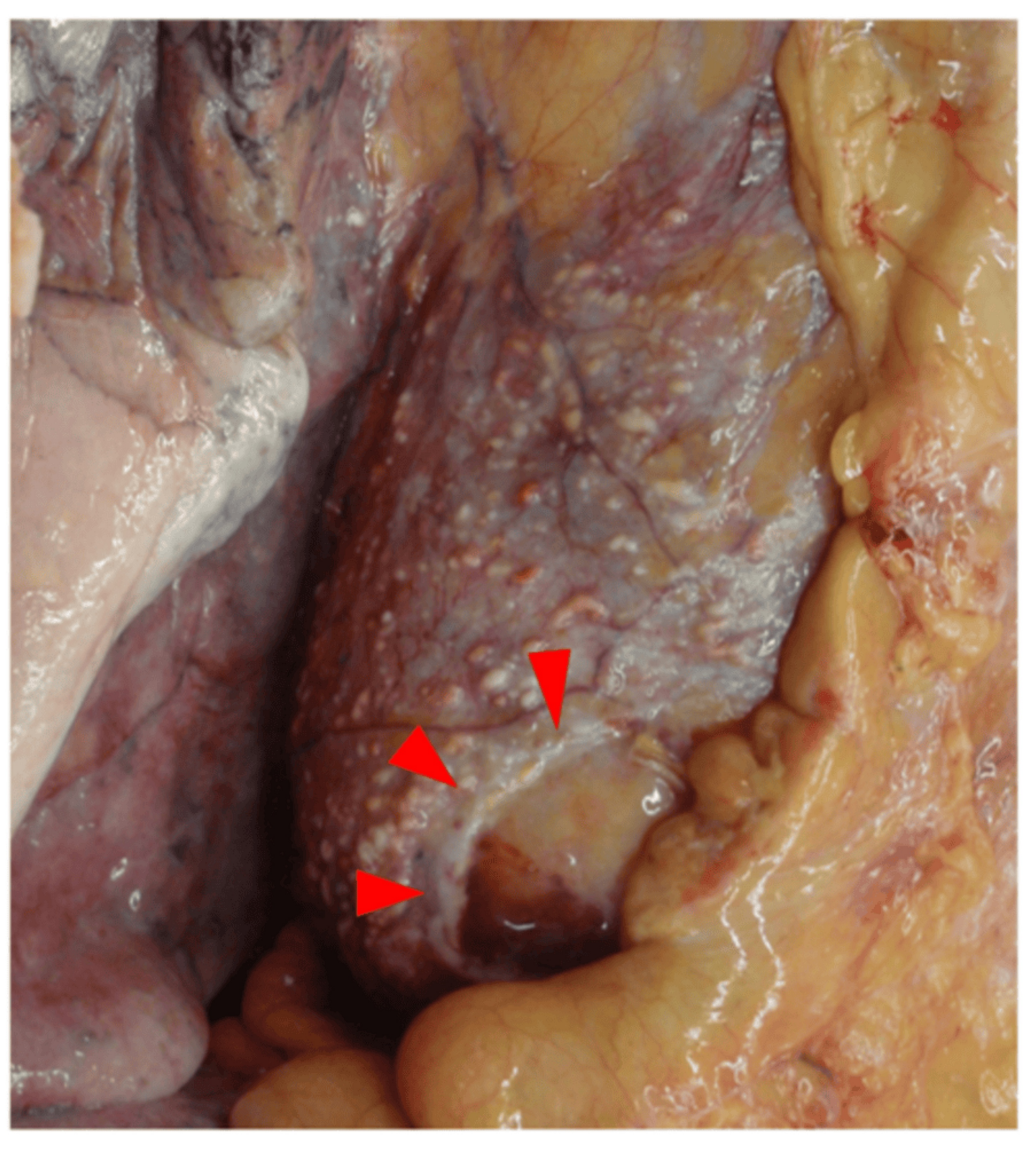
Autopsy findings. Dense adhesions surrounding the fenestration site (red arrows), resulting in compartmentalization of the pericardial cavity.

Autopsy further demonstrated dense adhesions around the fenestration site (Figure 2), which compartmentalized the pericardial cavity and resulted in localized reaccumulation.

## Discussion

This case highlights two clinically important lessons: (1) localized reaccumulation can occur even after technically adequate pericardial fenestration, and (2) such reaccumulation may be radiographically occult on standard frontal chest radiography.

First, localized reaccumulation can occur despite an apparently sufficient fenestration. In this case, a 3 × 3 cm pericardial window was created. Previous reports have shown that small fenestrations (e.g., 1 × 2 cm) without pleurodesis or pericardiodesis resulted in recurrence in three of five cases [6]. In contrast, patients with a large pericardial-peritoneal window (4 cm) achieved complete drainage, whereas recurrence occurred in a case with a smaller (3 cm) window [7]. Another study demonstrated the effectiveness of 2- to 4-cm pericardial resections [8]. Collectively, although no comparative trials directly address fenestration size, a 3 × 3 cm window was reasonable in this patient. Nevertheless, tumor dissemination and inflammatory responses due to a lack of effective systemic cancer control likely promoted adhesion formation, which obstructed the window and caused multiloculated effusion.

Second, reaccumulation after fenestration may not be apparent on frontal chest radiography. In our case, autopsy imaging revealed loculated effusions confined to the anterior and posterior pericardial spaces, rendering them invisible on frontal chest radiography. Generally, radiography provides only indirect evidence of pericardial effusion, and an enlarged cardiac silhouette typically requires at least 200 mL of fluid [9]. In this case, adhesions likely caused increased pericardial pressure with only a small amount of fluid, precipitating tamponade despite an apparently normal chest radiograph. Thus, recurrent tamponade may be overlooked if evaluation relies solely on chest radiography.

Echocardiography remains the primary diagnostic modality for pericardial effusion, with right atrial systolic collapse demonstrating 90% sensitivity and 68-82% specificity for tamponade [10]. In our case, echocardiography successfully identified tamponade before the fenestration, even though reaccumulation was localized to the anterior pericardial space and chest radiography appeared unremarkable. Moreover, echocardiography clearly showed pericardial effusion with hemodynamic compromise, whereas pulmonary embolism, pneumonia, and tumor progression were clinically excluded. The subsequent hemodynamic deterioration - marked tachycardia, hypotension, and refractory hypoxemia - corresponded well with recurrent cardiac tamponade rather than alternative causes of dyspnea. Additionally, contrast-enhanced CT is valuable when loculated effusion is suspected. For example, reflux of contrast medium into the azygos vein indicates significant hemodynamic compromise and, in the presence of pericardial effusion, suggests tamponade [11].

Therefore, in patients with postoperative respiratory deterioration after fenestration, multimodal imaging, including lateral chest radiography, echocardiography, or CT, should be pursued even when frontal chest radiography appears unremarkable.

## Conclusions

We report a case of localized pericardial effusion reaccumulation after fenestration that was undetectable on frontal chest radiography. Autopsy demonstrated dense adhesions around the fenestration site, which compartmentalized the pericardial cavity and resulted in localized reaccumulation. Multimodal imaging, particularly echocardiography and CT, should be considered in patients with unexplained respiratory deterioration following pericardial fenestration.
